# Exhaled Breath Analysis Using Selected Ion Flow Tube Mass Spectrometry and Disease Severity in Heart Failure

**DOI:** 10.3390/metabo13101049

**Published:** 2023-10-03

**Authors:** Wai Hong Wilson Tang, Lily Tranchito, Chonyang Albert, Zeynep G. Gul, Frank S. Cikach, David Grove, Yuping Wu, Raed A. Dweik

**Affiliations:** 1 Heart Vascular and Thoracic Institute, Cleveland Clinic, Cleveland, OH 44195, USA; 2 Endocrinology & Metabolism Institute, Cleveland Clinic, Cleveland, OH 44195, USA; 3 Department of Surgery, Washington University School of Medicine at St Louis, St Louis, MO 63110, USA; 4 Department of Inflammation and Immunology, Lerner Research Institute, Cleveland Clinic, Cleveland, OH 44195, USA; groved2@ccf.org (D.G.); dweikr@ccf.org (R.A.D.); 5 Respiratory Institute, Cleveland Clinic, Cleveland, OH 44195, USA; 6 Department of Mathematics, Cleveland State University, Cleveland, OH 44195, USA

**Keywords:** exhaled breath, selected ion flow tube mass spectrometry, acetone, pentane, heart failure

## Abstract

Exhaled breath volatile organic compounds (VOCs) are elevated in heart failure (HF). The ability of VOCs to predict long term cardiovascular mortality and morbidity has not been independently verified. In 55 patients admitted with acute decompensated heart failure (ADHF), we measured exhaled breath acetone and pentane levels upon admission and after 48 h of diuresis. In a separate cohort of 51 cardiac patients undergoing cardiopulmonary exercise testing (CPET), we measured exhaled breath acetone and pentane levels before and at peak exercise. In the ADHF cohort, admission acetone levels correlated with lower left ventricular ejection fraction (LVEF, r = −0.297, *p* = 0.035). Greater weight loss with diuretic therapy correlated with a greater reduction in both acetone levels (r = −0.398, *p* = 0.003) and pentane levels (r = −0.309, *p* = 0.021). In patients with above-median weight loss (≥4.5 kg), patients demonstrated significantly greater percentage reduction in acetone (59% reduction vs. 7% increase, *p* < 0.001) and pentane (23% reduction vs. 2% reduction, *p* = 0.008). In the CPET cohort, admission acetone and pentane levels correlated with higher VE/VCO2 (r = 0.39, *p* = 0.005), (r = 0.035, *p* = 0.014). However, there were no significant correlations between baseline or peak exercise acetone and pentane levels and peak VO2. In longitudinal follow-up with a median duration of 33 months, patients with elevated exhaled acetone and pentane levels experienced higher composite adverse events of death, ventricular assist device implantation, or orthotopic heart transplantation. In patients admitted with ADHF, higher exhaled breath acetone levels are associated with lower LVEF and poorer outcomes, and greater reductions in exhaled breath acetone and pentane tracked with greater weight loss. Exhaled acetone and pentane may be novel biomarkers in heart failure worthy of future investigation.

## 1. Introduction

Congestive heart failure (CHF) is a complex disease state that reflects inadequate cardiac perfusion and maladaptive activation of hormonal systems that further perpetuate congestion, tissue malperfusion, and metabolic disarray. Although biomarkers such as B-type natriuretic peptide and troponins have emerged as markers of myocardial stretch and injury, biomarkers of myocardial metabolism that reflect cellular and tissue characteristics in CHF are still in nascency. Patients with CHF have abnormalities in their heart structures and functions, causing their hearts to be unable to efficiently pump or fill adequately to meet their bodies’ metabolic demands. This can impact end organ function, causing fluid retention (in the form of weight gain and congestion) due to kidney dysfunction or poor exercise performance when directly measured using standardized cardiopulmonary exercise testing (CPET). The exhaled breath contains a mixture of hundreds of organic molecules which reflect the metabolic state of the individual and could be harnessed for diagnostic and therapeutic potential. Recent studies have shown that the levels of volatile organic compounds (VOCs) in exhaled breath correlate with certain disease states including CHF. Several studies have identified elevated levels of nitric oxide, pentane, and acetone in the exhaled breath in patients with CHF [1,2,3,4,5]. Data published from our own group has also shown the feasibility of measuring inpatient breath VOC analysis using selected ion flow tube mass spectrometry (SIFT-HF) technology, and the ability of exhaled acetone and pentane to discriminate between patients with and without CHF [6,7,8]. However, most exhaled breath studies to date have been case–control, cross-sectional analyses, and few examined how patients’ levels of exhaled VOCs correlate with their clinical changes or functional capacity. We sought to prospectively investigate exhaled acetone and pentane profiles in two patient populations: individuals admitted with diagnosis of acute decompensated heart failure (ADHF), and in outpatient individuals undergoing CPET. In the ADHF cohort, we sought to further define how the levels of VOCs changed with clinical status during a patient’s hospital stay, whereas in the CPET population, we aimed to explore the relationship between exhaled breath analytes with cardiopulmonary exercise physiology. In both cohorts of patients, we evaluated the long-term prognostic significance of VOCs.

## 2. Materials and Methods

### 2.1. Study Population

This is a single-center, prospective clinical study of patients admitted to the Cleveland Clinic between July 2012 and July 2013. Eligible patients were admitted with a diagnosis of ADHF and hypervolemia (ADHF cohort). The ADHF cohort represents patients admitted to the hospital with worsening signs and symptoms due to increasing congestion that requires diuresis (fluid removal by intravenous furosemide either using bolus or continuous administration). Weight change within 48 h of diuresis represents a surrogate measure of fluid removal for the treatment of edema and transition from decompensated to compensated state. The dosage and frequencies of administrations were determined at the discretion of the treating clinicians and blinded from the exhaled breath analysis. In addition, we included patients with a diagnosis of heart failure and/or cardiomyopathy undergoing clinically indicated CPET for symptomatic evaluation between September 2013 and March 2014 (CPET cohort). The CPET cohort represents patients with chronic heart failure undergoing an exercise challenge to quantify the degree of their cardiopulmonary reserve as a standard metric of disease severity by assessing oxygen and carbon dioxide exhalation. In both cohorts, we included patients who have a history of heart failure or cardiomyopathy presenting in these two clinical settings, and excluded patients who were unable to perform the exhaled breath analysis maneuvers or patients who were active cigarette smokers. These subjects were followed using electronic health record reviews for adverse events including death, VAD implantation, and OHT until May 2017, censored at the last known follow-up date.

### 2.2. Study Design

The study protocol was approved by the Cleveland Clinic Institutional Review Board and all patients were provided written informed consent. Upon enrollment, detailed clinical data were obtained through a review of the patients’ electronic medical records. Labs collected upon admission in the ADHF cohort, including NT-proBNP, sodium, transaminases, and estimated glomerular filtration rate (eGFR), were recorded. Pulmonary capillary wedge pressures and right atrial pressures were obtained from data collected during the course of hemodynamically guided treatment with pulmonary artery catheter placement in a dedicated heart failure intensive care unit. Baseline LVEF and LVIDd were collected from the patients’ most recent echocardiograms performed within 18 months for both cohorts. For the CPET cohort, cardiopulmonary exercise test data including peak oxygen consumption (peak VO2) and VE/VCO2 were collected as part of the clinical study using a modified Naughton protocol as previously described [9] (Ultima™ CardiO2, MGC Diagnostics, St Paul, MN, USA) during which the exhaled breath samples were collected.

### 2.3. Exhaled Breath Collection

Baseline ADHF exhaled breath samples were collected upon admission and follow-up breath samples were collected after at least 48 h of the baseline. Baseline metabolic stress lab exhaled breath samples were collected prior to exercise testing and follow-up breath samples were collected within 5 min of completion of CPET. All exhaled breath samples were collected following at least a 4 h fast, and bottled water mouth rinse was used to minimize and standardize the contribution of the aero-digestive tract. Breath samples were collected using a breath gauge, which consisted of a one-time-use sterile mouthpiece, an end filter to prevent ambient air contribution, a gauge to measure expiratory airflow, and a one-way valve that connected to the Mylar^®^ bag. Recruited patients were instructed to provide a single exhaled tidal volume into the sterile mouthpiece. The breath volume was collected while attempting to maintain an exhaled pressure of 15 millibars. Exhaled breath sample bags were stored in an incubator at 37 °C and processed within 4 h. The Mylar^®^ bags were flushed with nitrogen between uses.

### 2.4. Volatile Organic Compounds Testing Using Mass Spectrometry

All exhaled breath analyses were performed using SIFT-MS (Voice200, Syft Technologies, Christchurch, New Zealand) as previously described [1]. We used 3 precursor ions (H3O^+^, O2^+^, and NO^+^) one at a time to ionize the breath molecules. Twenty-two VOCs were quantified, including acrylonitrile and benzene, which are two VOCs without a known endogenous source, thus these two compounds were used as ambient controls as previously described [1,10]. In addition, VOCs previously described as elevated in the context of renal and/or liver impairment, such as ammonia, were quantified to control for the confounding effects of significant renal or liver dysfunction. Exhaled acetone and pentane were monitored using the product ions in Table 1.

### 2.5. Statistical Analysis

VOCs were visually assessed for normality. Comparison of parametric continuous variables was performed using Student’s *t*-test, non-parametric continuous variables were compared using the Wilcoxon Signed Rank Test, and categorical variables were compared using the Chi-square test. Kaplan–Meier analysis was performed in a pooled analysis of the two groups stratified by baseline exhaled acetone and exhaled pentane levels at the optimal cut-off value determined using receiver operator characteristic (ROC) curves (using survival ROC package, version 1.0.3). All analyses were performed using JMP Pro version 9.0 (SAS Institute, Cary, NC, USA) and R version 8.02 (Vienna, Austria).

## 3. Results

### 3.1. Patient Characteristics and Exhaled VOC Levels

Out of 97 ADHF patients, 55 had serial exhaled VOC levels collected, and among them 19 received right heart catheterization in the context of routine clinical care. Baseline characteristics of the ADHF patient populations are summarized in Table 2. In the ADHF cohort, baseline median exhaled acetone and pentane levels were 190.5 μg/L (interquartile ranges (IQR) 67.4 μg/L to 443.3 μg/L) and 17.2 μg/L (IQR 10.6 μg/L to 25.1 μg/L), respectively.

**Figure 1:**
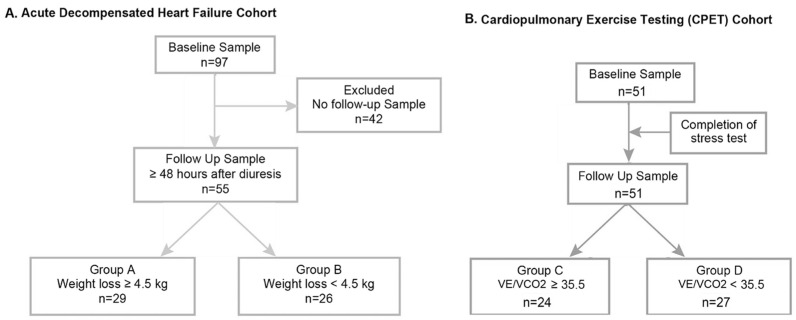
STROBE diagram of the study populations. Participants with heart failure were enrolled in two cohorts. In the acute decompensated heart failure cohort (**A**), hospitalized patients with decompensated heart failure were stratified according to those with (Group A) versus without (Group B) significant weight loss (cut-off at median weight loss of 4.5 kg over 48 h after diuresis). Exhaled breath samples were collected at baseline (within 24 h of admission) and after ≥48 h of diuresis. In the cardiopulmonary exercise testing (CPET) cohort (**B**), ambulatory patients with chronic heart failure were stratified according to those with (Group C) versus without (Group D) significant impairment in ventilatory efficiency (VE/VCO2 at median cut-off of 35.5). Exhaled breath samples were collected before and immediately after CPET.

To determine if levels of exhaled VOCs changed with clinical indicators of ADHF, such as hypervolemia, the ADHF cohort was divided into two groups based on above versus below the median weight loss (−4.5 kg). Group A consisted of 29 patients who lost at least 4.5 kg following diuresis, while Group B consisted of 26 patients who did not lose more than the median weight loss (Figure 1A). Group A (weight loss ≥4.5 kg) demonstrated numerically higher baseline median exhaled acetone levels (254 μg/L (IQR 126 μg/L to 460 μg/L) vs. 130 μg/L (IQR 44 μg/L to 441 μg/L), *p* = 0.073) and exhaled pentane levels (19.8 μg/L (IQR 12.4 μg/L to 24.6 μg/L) vs. 13.8 μg/L (IQR 8.2 μg/L to 25.7 μg/L), *p* = 0.159) when compared with Group B (weight loss < 4.5 kg) but neither reached statistical significance. There were no significant differences between the ADHF groups in age, body mass index, hypertension, admission aminoterminal pro-B-type natriuretic peptide (NT-proBNP) levels, left ventricular ejection fraction (LVEF), left ventricular end-diastolic diameter (LVIDd), or several co-morbidities theorized to result in alterations in the exhaled metabolome (i.e., diabetes mellitus, chronic obstructive pulmonary disease). There was a significant difference in pulmonary capillary wedge pressures (PCWP) between the groups, with those who experienced more weight loss after diuresis having higher median PCWP levels at baseline compared to those with less weight loss (Table 1).

Baseline characteristics of the CPET cohort are summarized in Table 2. In the CPET cohort of 51 patients, the baseline median exhaled acetone level was 158.8 μg/L (IQR 72.1 μg/L to 421.4 μg/L) and the baseline exhaled pentane level was 13.6 μg/L (IQR 10.9 μg/L to 19.5 μg/L). To determine if the levels of exhaled VOCs correlated with clinical indicators of heart failure severity, the CPET cohort was divided into two groups based on above versus below the median minute ventilation/carbon dioxide production slope (VE/VCO2) level (35.5). Group C consisted of patients who had a VE/VCO2 ≥35.5, while Group D consisted of those who had a VE/VCO2 measurement below the median level (Figure 1B). Besides age, there were no significant differences between metabolic stress lab groups in body mass index, hypertension, or several co-morbidities that may affect exhaled metabolome (Table 3). None of the participants were smokers. We observed no differences in VOCs when stratified according to the median peak oxygen consumption (peak VO2).

### 3.2. Acute Changes in VOC Following Diuresis

**Figure 2:**
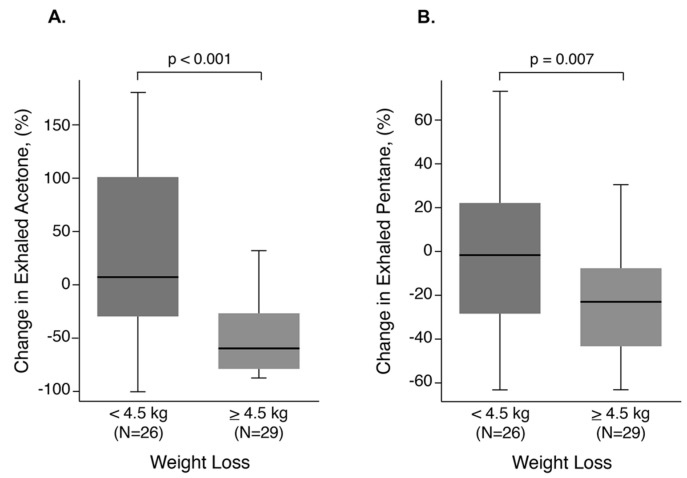
Changes in exhaled acetone/pentane and weight loss in the ADHF cohort. Changes in exhaled acetone (**A**) and exhaled pentane (**B**), stratified by median weight loss (−4.5 kg) following diuresis in patients admitted for acute decompensated heart failure (ADHF).

After ≥48 h of diuresis, the median exhaled acetone and exhaled pentane levels were reduced to 103.5 μg/L (IQR 49.6 μg/L to 245.4 μg/L) and 12.2 μg/L (IQR 10.2 μg/L to 21.0 μg/L), respectively. Group A demonstrated numerically lower median levels of post-diuresis exhaled acetone (70.5 μg/L (IQR 52.1 μg/L to 324.7 μg/L) vs. 157.5 μg/L (IQR 48.5 μg/L to 272.1 μg/L), *p* = 0.484) and exhaled pentane levels (11.8 μg/L (IQR 9.8 μg/L to 20.2 μg/L) vs. 13.1 μg/L (IQR 10.6 μg/L to 21.4 μg/L), *p* = 0.484)) compared to Group B, but neither reached statistical significance. However, when analyzed according to changes from baseline to follow-up, there was a significantly greater percentage reduction in acetone (59% reduction vs. 7% increase, respectively, *p* < 0.001) and pentane (23% reduction vs. 2% reduction, respectively, *p* = 0.008) between Groups A and B (Figure 2). In contrast, there was no significant difference in the percent changes in exhaled ammonia, acrylonitrile, and benzene between the two groups.

**Figure 3:**
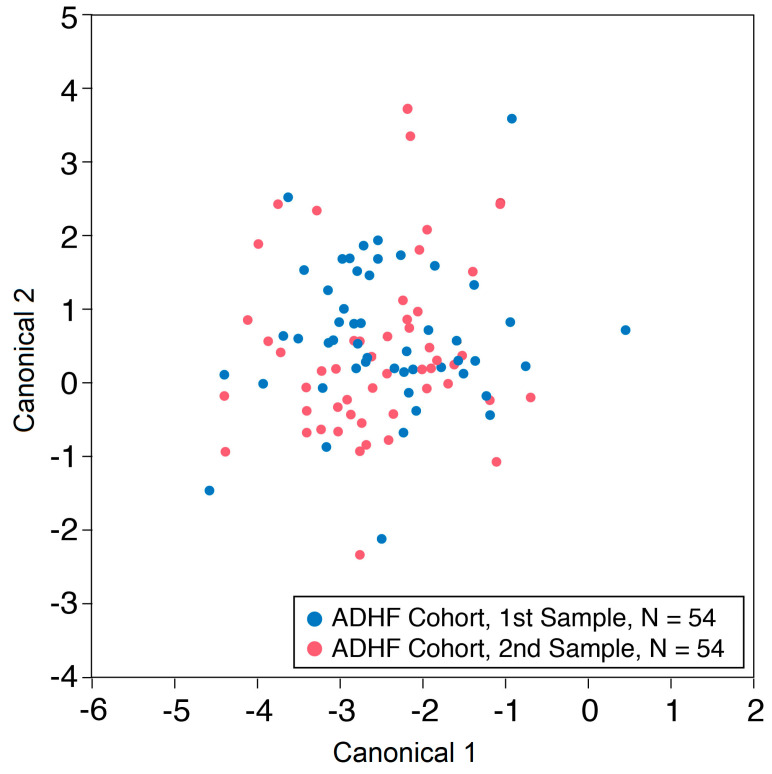
Canonical discriminant analysis breathprint patterns. Comparison of breathprint patterns between baseline and follow-up exhaled breath samples. In the ADHF cohort of 55 participants, one individual was excluded with lack of corresponding follow-up breathprint.

Furthermore, we observed no discernable differences in the breathprint patterns before and after diuresis (Figure 3).

### 3.3. Acute Changes in VOC Following Exercise

In the CPET cohort, the post-exercise median exhaled acetone level was increased to 183.2 μg/L (IQR 69.0 μg/L to 480.3 μg/L) and the post-exercise median exhaled pentane was increased to 13.6 μg/L (IQR 10.9 μg/L to 19.5 μg/L). Group C (VE/VCO2 ≥35.5) demonstrated significantly greater median baseline exhaled acetone levels (212.0 μg/L (IQR 124.0 μg/L to 594.9 μg/L) vs. 117.2 μg/L (IQR 47.5 μg/L to 291.4 μg/L), *p* = 0.034) and baseline exhaled pentane levels (16.0 μg/L (IQR 11.6 μg/L to 26.2 μg/L) vs. 11.7 μg/L (IQR 10.3 μg/L to 15.3 μg/L), *p* = 0.049) when compared with Group D (VE/VCO2 < 35.5). Group C also demonstrated significantly greater median post-exercise exhaled acetone levels compared to Group D (278.7 μg/L (IQR 129.9 μg/L to 703.2 μg/L) vs. 111.8 μg/L (IQR 33.0 μg/L to 356.5 μg/L), *p* = 0.049), but no significant difference was demonstrated in the median post-exercise exhaled pentane levels between the two groups (18.5 μg/L (IQR 11.6 μg/L to 26.6 μg/L) vs. 14.4 μg/L (IQR 10.0 μg/L to 18.2 μg/L), *p* = 0.093). When analyzed according to changes from baseline to post-exercise levels, there were no statistically significant differences in percentage reduction in exhaled acetone (7.5% increase vs. 6.9% increase, *p* = 0.900) or exhaled pentane (2.7% increase vs. 3.2% increase, *p* = 0.992) between Groups C and D. Again, there was no significant difference in the percent changes in exhaled ammonia, acrylonitrile, and benzene between the two groups.

### 3.4. Relationship between Prespecified VOCs and Hemodynamic and Cardiopulmonary Exercise Parameters

In the ADHF cohort, there was a significant negative correlation between higher baseline exhaled acetone levels and lower LVEF (Spearman’s r = −0.29, *p* = 0.035). In contrast, there were no significant correlations between baseline exhaled acetone or exhaled pentane levels and pulmonary capillary wedge pressure (PCWP), cardiac index, LVIDd, NT-proBNP, body mass index, or age. Also, baseline pharmacotherapy with beta-blockers, angiotensin converting enzyme inhibitors, statins, or insulin did not have demonstrable effects on these VOCs. In the CPET cohort, baseline exhaled acetone and exhaled pentane levels correlated with higher VE/VCO2 (r = 0.39, *p* = 0.005), (r = 0.035, *p* = 0.014). In contrast, there was no significant correlation between baseline exhaled acetone and exhaled pentane levels and peak VO2.

### 3.5. Relationship between Exhaled VOC and Long-Term Outcomes

**Figure 4:**
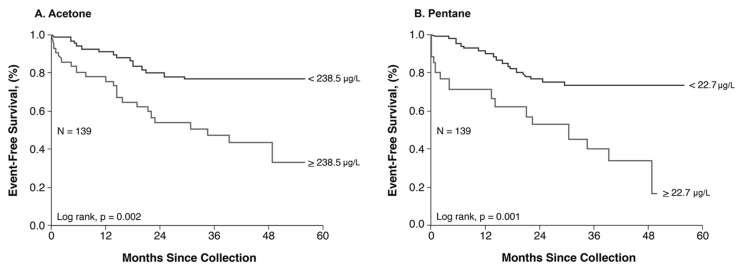
Major adverse clinical outcomes stratified by median baseline exhaled acetone and exhaled pentane levels. Cut-off values for exhaled acetone (238.5 μg/L, (**A**)) and pentane (22.7 μg/L, (**B**)) determined using the best optimal cut-off value determined using receiver operator characteristic (ROC) curves in the pooled cohort (*n* = 139).

In our ADHF cohort, we observed a 38% mortality rate with 7% of the patients receiving ventricular assist devices (VAD), and 11% receiving orthotopic heart transplantation (OHT). In our CPET cohort, we observed a 16% mortality rate with 2% of the patients receiving VAD, and 6% receiving OHT. Longitudinal follow-up of both ADHF and CPET groups was available in 139 subjects over a median follow-up period of 33 months and demonstrated increased adverse events with elevated exhaled acetone (Log-rank, *p* = 0.002) and exhaled pentane (Log-rank, *p* = 0.001, Figure 4).

## 4. Discussion

### 4.1. Key Findings

We performed direct serial quantification of specific VOCs in an independent cohort of patients treated for ADHF using SIFT-MS technology with some key findings. First, we observed a greater reduction in exhaled levels of acetone and pentane in patients who achieved a greater net negative fluid balance or lost more weight following diuresis. In patients who were admitted with ADHF, those with greater weight loss following diuresis exhibited a greater decrease in exhaled levels of acetone and pentane. These findings substantiated data reported by our group and others that implicated exhaled acetone and pentane as predictive of response to decongestion therapy. Second, in an independent ambulatory cohort undergoing cardiopulmonary exercise testing we observed a direct correlation between exhaled acetone and pentane levels and estimates of functional capacity as measure of disease severity. In patients who underwent metabolic stress lab testing, there were higher baseline acetone and exhaled pentane levels in those who had higher VE/VCO2 measurements during testing. Third, we demonstrated that changes in exhaled levels of acetone and pentane correlated with changes in weight following decongestion in this cohort of patients admitted for ADHF. In addition, we demonstrated the ability of exhaled acetone and exhaled pentane as markers of long-term prognosis in disease-free survival. However, we did not observe any clear relationship between changes in hemodynamic derangements or peak oxygen consumption and exhaled acetone and exhaled pentane levels, nor did we observe a shift in the unique heart failure “breathprint” as previously described. Taken together, our findings further support the clinical relevance for profiling exhaled gas patterns in risk stratification of patients with heart failure. In determining the biomarker potential of exhaled VOCs, our findings demonstrate the feasibility of collecting inpatient and outpatient serial quantifications of VOCs, the ability of VOCs to predict response to decongestive therapy, and the ability of VOCs to prognosticate future disease-free survival.

### 4.2. Exhaled Acetone and Pentane in Heart Failure

This study also confirms that changes in exhaled levels of acetone and pentane correlate with changes in disease severity of ADHF patients, as suggested by several previous reports [2,3,4,5,6,7,8,11,12,13] and demonstrates the feasibility of using single exhaled breath samples for risk stratification in ADHF. A recently pilot CPET study in HF patients also showed increased breath levels of acetone and pentane at rest using SIFT-MS technology [2]. The fact that there were no changes in ammonia levels on serial quantification suggested that the exhaled metabolomics profile in ADHF is not simply a reflection of baseline differences in end-organ function. Acetone is synthesized in the liver by the decarboxylation of excess acetyl-CoA via fatty acid β-oxidation. Exhaled breath acetone has previously been studied as a possible marker of disease severity for patients with ADHF. Kupari et al. observed that patients who experienced the most severe systemic venous congestion produced the highest breath acetone levels [14]. HF patients with more prominent jugular vein distention (JVD) (≥5 cm) exhaled, on average, a breath acetone level four times higher than those with a JVD of less than 5 cm [14]. Marcondes-Braga et al. showed that the median concentration of exhaled acetone was significantly higher in ADHF patients than in chronic HF patients [15]. They also found that as the severity of HF increased, as measured using the NYHA functional class, there was a significant increase in exhaled acetone levels [15]. We improved on their study design in two ways. Instead of gas chromatography–mass spectrometry, we utilized SIFT-MS, a newer technology that does not require sample pre-concentration which allows for real-time analysis of the breath samples. In addition, we did not exclude patients with diabetes and renal failure, making our results more clinically relevant. On the other hand, pentane, a result of lipid peroxidation and oxidative stress, is considered an important biomarker of many metabolic processes and inflammation [16]. Lipid peroxidation has been shown to increase proportionally with the severity of heart failure [17], and therefore exhaled levels of pentane could potentially be used as a non-invasive disease monitoring technique. Our previous study supported the theory that inflammation is indicated by exhaled pentane levels [1].

### 4.3. Clinical Implications

Serial sampling allowed us to monitor how VOC levels change within individuals as they improve clinically. Our results demonstrate the possibility of analyzing exhaled breath samples as a disease monitoring technique. SIFT-MS has mitigated cumbersome sample preparation and processing, allowing exhaled breath analysis to be performed in real time in the hospital setting. Like other clinically applicable exhaled breath measurements, the true promise lies in the potential for point-of-care and ambulatory monitoring and screening. Once clinically relevant VOCs are identified, highly sensitive and specific solid-state sensors can be integrated into portable detectors.

As with all novel diagnostic tests, the evolution from conceptualization to implementation is a lengthy process, particularly when the platform in question is a radical departure from standard body fluid analysis. For example, consensus guidelines for the use of exhaled nitric oxide in the assessment of patients with reactive airway disease did not emerge until approximately two decades after the initial demonstration of altered levels in the breath [18]. Future studies in exhaled breath metabolomics are necessary to accelerate the progress in the field of cardiovascular medicine.

### 4.4. Study Limitations

Results of this study are limited by the small sample size, single center experience, and somewhat limited median follow-up of 33 months. Larger prospective studies are necessary to validate these results. In all studies utilizing exhaled gas analysis there are technical uncertainties that must be acknowledged, like the volatility of exhaled breath, which makes these techniques highly susceptible to clinical confounders, as well as the timing and context of data collection. While we did not identify consistent pharmacotherapy-induced alterations in exhaled VOCs, this may have been related to insufficient power in this hypothesis-generating study. It is almost certain that there are important alterations in the exhaled metabolome related to pharmacotherapy. At the tissue level, divergent metabolic profiles have been demonstrated with even the most subtle variations in exposure, for example to different enantiomers of carvedilol [19]. Finally, formal methods for the analysis of metabolomic data (e.g., data reduction techniques) are still evolving and need to be firmly established for future studies. However, studies within our lab demonstrate that while intra-patient coefficients of variation of <2% were achieved when multiple measurements were obtained within a single sitting, fasting morning measurements 10 days apart demonstrated an intra-individual coefficient of variation of 15%. The variation increased to 26% when observing intra-day measurements (i.e., morning vs. afternoon). These variations highlight the importance of establishing biological and assay variability for each VOC studied and understanding the impact of diurnal variations, concomitant pharmacotherapy, comorbid disease states, and the feeding/fasting state. Since the exhaled VOC samples for each patient were collected in the same room (inpatient room for ADHF cohort at different days and CPET laboratory in the CPET cohort within the same day), environmental influence should be minimal.

## 5. Conclusions

In patients admitted with ADHF, higher exhaled breath acetone levels are associated with lower LVEF, and greater reductions in exhaled breath acetone and pentane are correlated with greater weight loss. Furthermore, higher pre- and post-exercise exhaled acetone and pre-exercise exhaled pentane levels are associated with higher VE/VCO2 but not peak VO2 in stable cardiac patients undergoing CPET. Additionally, longitudinal follow-up demonstrates the ability of VOCs to discriminate patients with respect to risk of cardiovascular morbidity and mortality including VAD, OHT, and death. In combination, this constellation of findings supports the role of VOCs as potential biomarkers in heart failure diagnostics, therapeutics, and prognostication.

## Figures and Tables

**Table 1:** Quantification of volatile organic compounds testing using mass spectrometry.

Metabolite	Precursor	Product Mass (*m*/*z*)	Ions Measured	Branching Ratio
Acetone	H3O+	59	C_3_H_7_O+	100%
	NO+	88	NO+*C_3_H_6_O	100%
Pentane	O2+	42	C_3_H_6_+	40%
		72	C_5_H_12_+	10%

**Table 2:** Baseline characteristics of patients in the acute decompensated heart failure cohort.

Characteristics	Total Population (*n* = 55)	Weight Loss ≥4.5 kg (*n* = 29)	Weight Loss <4.5 kg (*n* = 26)	*p*-Value
Demographics				
Age (years)	65 (54, 70)	65 (55, 69)	64 (52, 70)	0.717
Male, *n* (%)	37 (67.3)	19 (65.5)	18 (69.2)	0.996
BMI (kg/m^2^)	30.6 (25.9, 35.9)	33.8 (27.4, 37.3)	29.8 (25.1, 33.4)	0.079
Comorbidities				
Hypertension, *n* (%)	33 (60)	18 (62.1)	15 (57.7)	0.956
Diabetes mellitus, *n* (%)	29 (52.7)	17 (58.6)	12 (46.2)	0.513
Coronary artery disease, *n* (%)	31 (56.4)	16 (55.2)	15 (57.7)	1.000
Atrial fibrillation, *n* (%)	32 (59.3)	17 (60.7)	15 (57.7)	1.000
COPD, *n* (%)	11 (20)	4 (13.8)	7 (26.9)	0.380
Laboratory data				
NT-pro BNP (pg/mL)	4226 (1726, 8627)	4408 (1752, 5809)	3018 (1654, 10,980)	0.936
eGFR (mL/min/1.73 m^2^)	45 (29, 61)	45 (32, 56)	45 (24, 68)	0.952
Sodium (mmol/L)	139 (136, 140)	139 (136, 141)	138 (135, 140)	0.451
AST (U/L)	28 (19, 35)	22 (17, 37)	30 (23, 35)	0.202
ALT (U/L)	21 (16, 30)	18 (15, 28)	24 (19, 30)	0.062
Echocardiographic data				
LVEF (%-units)	36 (20, 50)	35 (19, 48)	38 (24, 57)	0.367
LVIDd (mm)	52.5 (45, 62.2)	56 (46.5, 65)	51 (44.2, 61)	0.272
Hemodynamic data				
PCWP (mmHg)	27 (24, 30)	30 (30, 34)	24 (22, 25)	0.004 *
RAP (mmHg)	16 (12, 21)	19 (14, 22)	15 (11, 18)	0.161
Medications				
ACE-i/ARB, *n* (%)	25 (49)	15 (53.6)	10 (43.5)	0.663
β-blocker, *n* (%)	44 (86.3)	27 (96.4)	17 (73.9)	0.055
Statin, *n* (%)	32 (64)	20 (74.1)	12 (52.2)	0.189
Loop diuretic, *n* (%)	47 (92.2)	26 (92.9)	21 (91.3)	1.000
Aspirin, *n* (%)	36 (75)	22 (81.5)	14 (66.7)	0.401
Insulin, *n* (%)	15 (29.4)	9 (32.1)	6 (26.1)	0.870

Caption: Baseline characteristics of patients admitted with acute decompensated heart failure stratified according to median weight loss of 4.5 kg after ≥48 h of diuresis, representing greater (≥4.5 kg) vs. lesser (<4.5 kg) treatment response to intravenous diuretics for relieving congestion. Non-parametric variables expressed as median (interquartile ranges) unless specified. * Abbreviations: BMI = body mass index (metric of obesity); COPD = chronic obstructive pulmonary disease; NT-proBNP = aminoterminal pro-B-type natriuretic peptide (metric of heart failure severity); eGFR = estimated glomerular filtration rate (metric of kidney function); AST = aspartate transaminase (metric of liver function); ALT = alanine transaminase (metric of liver function); LVIDd = left ventricular internal diameter at diastole (metric of cardiac dilation); LVEF = left ventricular ejection fraction (metric of cardiac function); PCWP = pulmonary capillary wedge pressure (metric of left-side filling pressure); RAP = right atrial pressure (metric of right-side filling pressure); ACE-I = angiotensin converting enzyme inhibitor; ARB = angiotensin receptor blocker.

**Table 3:** Baseline characteristics of patients in the cardiopulmonary exercise testing cohort.

Characteristics	Total Population (*n* = 51)	VE/VCO2 ≥ 35.5 (*n* = 24)	VE/VCO2 < 35.5 (*n* = 27)	*p*-Value
Age (years)	59 (50, 64)	62 (58, 66)	56 (46, 60)	0.007 *
Male, *n* (%)	36 (70.6)	19 (79.2)	17 (63)	0.337
BMI (kg/m^2^)	29.7 (27.1, 34.1)	30 (27.3, 33.7)	29.2 (26.9, 34.1)	0.928
Hypertension, *n* (%)	30 (60)	17 (70.8)	13 (50)	0.225
Diabetes mellitus, *n* (%)	12 (24)	8 (33.3)	4 (15.4)	0.249
Coronary artery disease, *n* (%)	20 (40)	13 (54.2)	7 (26.9)	0.094
Atrial fibrillation, *n* (%)	16 (32)	10 (41.7)	6 (23.1)	0.269
COPD, *n* (%)	3 (6)	1 (4.2)	2 (7.7)	1
LVEF (%-units)	32 (25, 60)	25 (25, 44)	51 (23, 64)	0.359

Caption: Baseline characteristics of ambulatory patients undergoing cardiopulmonary exercise testing according to median VE/VCO2 cut-off at 35.5, representing preserved (<35.5) versus impaired (≥35.5) ventilatory efficiency as a metric of disease severity. Non-parametric variables expressed as median (interquartile ranges) unless specified. * Abbreviations: VE/VCO2 = minute ventilation required to eliminate carbon dioxide (metric of ventilatory efficiency); BMI = body mass index (metric of obesity); COPD = chronic obstructive pulmonary disease; LVEF = left ventricular ejection fraction (metric of cardiac function).

## Data Availability

The data that support the findings of this study are available from the corresponding author upon reasonable request. The data are not publicly available due privacy.

## References

[1] Samara M.A., Tang W.H., Cikach F., Gul Z., Tranchito L., Paschke K.M., Viterna J., Wu Y., Laskowski D., Dweik R.A. (2013). Single exhaled breath metabolomic analysis identifies unique breathprint in patients with acute decompensated heart failure. J. Am. Coll. Cardiol..

[2] Biagini D., Pugliese N.R., Vivaldi F.M., Ghimenti S., Lenzi A., De Angelis F., Ripszam M., Bruderer T., Armenia S., Cappeli F. (2023). Breath analysis combined with cardiopulmonary exercise testing and echocardiography for monitoring heart failure patients: The AEOLUS protocol. J. Breath. Res..

[3] Gouzi F., Ayache D., Hedon C., Molinari N., Vicet A. (2021). Breath acetone concentration: Too heterogeneous to constitute a diagnosis or prognosis biomarker in heart failure? A systematic review and meta-analysis. J. Breath. Res..

[4] Marcondes-Braga F.G., Gioli-Pereira L., Bernardez-Pereira S., Batista G.L., Mangini S., Issa V.S., Fernandes F., Bocchi E.A., Ayub-Ferreira S.M., Mansur A.J. (2020). Exhaled breath acetone for predicting cardiac and overall mortality in chronic heart failure patients. ESC Heart Fail..

[5] Yokokawa T., Sato T., Suzuki S., Oikawa M., Yoshihisa A., Kobayashi A., Yamaki T., Kunii H., Nakazato K., Suzuki H. (2018). Change of Exhaled Acetone Concentration Levels in Patients with Acute Decompensated Heart Failure. Int. Heart J..

[6] Yokokawa T., Sato T., Suzuki S., Oikawa M., Yoshihisa A., Kobayashi A., Yamaki T., Kunii H., Nakazato K., Suzuki H. (2017). Elevated exhaled acetone concentration in stage C heart failure patients with diabetes mellitus. BMC Cardiovasc. Disord..

[7] Yokokawa T., Ichijo Y., Houtsuki Y., Matsumoto Y., Oikawa M., Yoshihisa A., Sugimoto K., Nakazato K., Suzuki H., Saitoh S.I. (2017). Change of Exhaled Acetone Concentration in a Diabetic Patient with Acute Decompensated Heart Failure. Int. Heart J..

[8] Biagini D., Lomonaco T., Ghimenti S., Bellagambi F.G., Onor M., Scali M.C., Barletta V., Marzilli M., Salvo P., Trivella M.G. (2017). Determination of volatile organic compounds in exhaled breath of heart failure patients by needle trap micro-extraction coupled with gas chromatography-tandem mass spectrometry. J. Breath. Res..

[9] American Thoracic Society, American College of Chest Physicians (2003). ATS/ACCP Statement on cardiopulmonary exercise testing. Am. J. Respir. Crit. Care Med..

[10] Smith D., Spanel P. (2005). Selected ion flow tube mass spectrometry (SIFT-MS) for on-line trace gas analysis. Mass. Spectrom. Rev..

[11] Yokokawa T., Sugano Y., Shimouchi A., Shibata A., Nakayama T., Ohara T., Jinno N., Kanzaki H., Anzai T. (2016). A case of acute decompensated heart failure evaluated by series of exhaled acetone concentrations as noninvasive biomarker of heart failure severity. Int. J. Cardiol..

[12] Yokokawa T., Sugano Y., Shimouchi A., Shibata A., Jinno N., Nagai T., Kanzaki H., Aiba T., Kusano K., Shirai M. (2016). Exhaled Acetone Concentration Is Related to Hemodynamic Severity in Patients with Non-Ischemic Chronic Heart Failure. Circ. J. Off. J. Jpn. Circ. Society..

[13] Marcondes-Braga F.G., Batista G.L., Gutz I.G., Saldiva P.H., Mangini S., Issa V.S., Ayub-Ferreira S.M., Bocchi E.A., Pereira A.C., Bacal F. (2016). Impact of Exhaled Breath Acetone in the Prognosis of Patients with Heart Failure with Reduced Ejection Fraction (HFrEF). One Year of Clinical Follow-up. PLoS ONE.

[14] Kupari M., Lommi J., Ventila M., Karjalainen U. (1995). Breath acetone in congestive heart failure. Am. J. Cardiol..

[15] Marcondes-Braga F.G., Gutz I.G., Batista G.L., Saldiva P.H., Ayub-Ferreira S.M., Issa V.S., Mangini S., Bocchi E.A., Bacal F. (2012). Exhaled acetone as a new biomaker of heart failure severity. Chest.

[16] Dryahina K., Španěl P., Pospíšilová V., Sovová K., Hrdlička L., Machková N., Lukáš M., Smith D. (2013). Quantification of pentane in exhaled breath, a potential biomarker of bowel disease, using selected ion flow tube mass spectrometry. Rapid Commun. Mass. Spectrom..

[17] Sobotka P.A., Brottman M.D., Weitz Z., Birnbaum A.J., Skosey J.L., Zarling E.J. (1993). Elevated breath pentane in heart failure reduced by free radical scavenger. Free Radic. Biol. Med..

[18] Kostikas K., Minas M., Papaioannou A.I., Papiris S., Dweik R.A. (2011). Exhaled nitric oxide in asthma in adults: The end is the beginning?. Curr. Med. Chem..

[19] Wang M., Bai J., Chen W.N., Ching C.B. (2010). Metabolomic Profiling of Cellular Responses to Carvedilol Enantiomers in Vascular Smooth Muscle Cells. PLoS ONE.

